# 

**Published:** 1829-04-01

**Authors:** 


### BIBLIOGRAPHICAL RECORD.

1. Comments on Corpulency, Lineaments of Leanness, Mems on Diet and Dietetics. By William Wadd, Esq. F.L.S. Surgeon extraordinary to the King, &c. 8vo. pp. 170. Christmas, 1828.

2. On Aneurism, and its Cure, by a New Operation. Dedicated by permission, to the King. By James Wardrop, Surgeon to His Majesty. 8vo. pp. 120, with numerous Plates. 1829.

3. An Elementary Compendium of Physiology; for the use of Students. By F. Magendie, M.D. &c. &c. &c. translated from the French, with copious Notes, Tables, and Illustrations, by E. Milligan, M.D. &c. &c. &c. Third Edition, with Alphabetical Index, Additional Notes, and Engravings, greatly enlarged. 8vo, pp. 618. Edinburgh and London, 1829, price 21s.

4. A Synopsis of Modern Medical Jurisprudence, Anatomically, Physiologically, and Forensically Illustrated; for the Faculty of Medicine, Magistrates, Lawyers,

Coroners, and Jurymen. By J. S. FORSYTH, Surgeon, &c. 12mo. pp. 600. London, 1829.

5. Traité Elementaire de l'Art des Accouchemens, ou Principes de Toxiologie, et d'Embryologie. Par ALF. A. M. VELPEAU, D.M.P. &c. &c. &c. Tomes 2. 8vo. pp. 962, à Paris, chez J. B. Bailliere, 1829.

6. The Medical Calendar; or, Student's Guide to the Medical Schools in Edinburgh, London, Dublin, Paris, Oxford, Cambridge, Glasgow, Aberdeen, and St. Andrews; together with the Regulations of the Public Boards, and Conditions of Admission into the Medical Corporations in Great Britain and Ireland. 8vo. pp. 220. 4s. Ed. 1829.

7. A Letter to the Rt. Honourable the Secretary of State for the Home Department, containing Remarks on the Report of the Select Committee of the House of Commons, on Anatomy, and pointing out the means by which the Science may be cultivated with advantage and safety to the public. By G. J. GUTHRIE, F.R.S. &c. &c. &c. 8vo. stitched, pp. 36. Sams, London, 1829. Price One Shilling.

8. Analytic Physiology, treating of the Cure of Nervous Diseases, by External Applications to the Spine. By SAM. HOOD, M.D., A.B. Second Edition, with an Appendix. 8vo. pp. 207, London, 1829.

9. A New S stem of Treating the Human Teeth; Explaining the Causes which lead to their Decay, and the most Approved Methods of Preserving them; with Copious and Explanatory Notes, to which is added some account of a Discovery made by the Author for the Cure of Tooth-Ache and Tic Douloureux, &c. By J. PATERSON CLARK, M.A. Dentist. 8vo. pp. 163, London, 1829.

10. The Influence of Physical Education in Producing and Confirming in Females, Deformity of the Spine. By E. W. DUFFIN, Surgeon. 8vo. pp. 135, London, 1829.

11. Traite d'Anatomie Pathologique. Par J. F. LOBSTEIN, Professeur de Clinique interne et d'Anatomie Pathologique à la Faculté de Medecine de Strasbourg. Directeur de son Musee Anatomique, &c. &c. a Paris. 8vo. pp. 568. 1829.

Avec, Six Planches Colorisees, en folio.

12. A Manual for the Use of Students preparing for Examination at Apothecaries' Hall. By JOHN STEGGALL, M.D. M R.C.S. &c. &c. 12mo. pp. 260, Anderson, 1829.

☞ *A very useful little work.*

13. A General, Medical, and Statistical History of the present condition of Public Charity in France; comprising a detailed account of all Establishments destined for the Sick, the Aged, and the Infirm, for Children and for Lunatics; with a view of the Extent of Pauperism and Mendicity, and the means now adopted for their Relief and Repression. By DAVID JOHNSTON, M.D. F.R.S.E. &c. &c. 8vo. pp. 605, Edinburgh, 1829.

14. The Manual for Invalids. By a Physician. 12mo. pp. 368, Edward Bull, London, 1828.

15. A Treatise on Diseases of the Chest, and on Mediate Auscultation. By R. T. H. LAENNEC, M.D. &c. &c. &c. Translated from the latest French edition, with Notes, and a Sketch of the Author's Life, by John Forbes, M.D. &c. 8vo. pp. 734. Third Edition revised, with Additional Notes. Underwoods', 1829.

16. A Series of Tables, exhibiting the Results of Disease in the different European Regiments serving under the Madras Presidency, &c. &c. 8vo, pp. 482. Madras. 1828.

17. A Treatise on Obstructed and Inflamed Hernia; and on Mechanical Obstructions of the Bowels Internally, and also an Appendix, containing a brief statement of the Cause of Difference in Size between the Male and Female Bladder. By HENRY STEPHENS, M.R.C.S. 8vo. pp. 191, London, 1829.

18. An Essay on the Use of Nitrate of Silver in the Cure of Inflammation, Wounds, and Ulcers. By JOHN HIGGINBOTTOM, &c. Second Edition enlarged and improved. 8vo. pp. 204, 1829.

19. A Manual of the Science and Art of Midwifery, &c. &c. By MICHAEL RYAN, M.D., &c. &c.

☞ *Dr. Ryan's Manual evinces considerable research, acuteness of observation, and talent. Fault may be found with the manner rather than with the matter of the work.*

20. Elements of Pathology and Practice of Physic. By JOHN MACKINTOSH, M.D. Acting Surgeon to the Ordnance in North Britain, &c. &c. &c. Vol. I. 8vo. pp. 484, Edinburgh and London, 1828.

### SUBSCRIBERS.

Bernard, Mr. Surgeon, Wells.
Bouragan, Dr. Chatterton.
Cairnie, Mr. Honble East India Cmopany's Service.
Chetculi, Dr. Thomas, Malta.
Chinnock, Mr. Michael's Place, Brompton.
Dewint, Mr. T. M.R.C.S. Ancaster Grantham, Lincolnshire.
Dill, J. Esq. Regency Square, Brighton.
Follcott, William, M.D. M.C.S.L. Clonakilty, County of Cork.
Foote, Dr. Baker Street, London.
Loudon, Dr. Leamington Spa, Warwickshire.
Macaan, Dr. Surg. 67th Foot, Liverpool.
M'Vickar, Dr. Benjamin, New York.
Ore, Assistant-surgeon, Malta.
Pettigrew, T. J. Esq. Saville Street.
Plymsoll, Mr. J. H. James Square, Edinburgh.
Roy, Dr. Park Street, Portman Square.
Smith, Mr. Surgeon, Great Haddam.

### INDEX.

A.

Abercrombie, Dr. on diseases of the stomach .................... 328
Abortion, Mad. Boivin on.......... 257
Abortion produced by mercury...... 472
Abscess in the substance of bone.... 410
Abscess of the liver, Mr. Annesley on 314
Abscess in the liver, Mr. Annesley on 435
Abscess in the cerebrum unsuspected 470
Abscess, insidious, in front of the larynx and trachea .................... 575
Absorbents, nitrate of silver in inflammation of .................... 446
Acute rheumatism, opiates in ...... 461
Addison, Mr. on the Malvern Waters 24
Adipocere, exostosis converted into! 157
Age of Libel.................... 266
Alison, Dr. on tubercles............ 72
Allan, Mr. his case of spinal neuralgia 244
Aloes, Battley's watery extract of.... 195
Amenorrhœa produced by mercury .. 471
Amputation of cervix uteri, M. Lisfranc on.................... 159
Amputation of the uterus .......... 215
Amputation, Mr. Guthrie on......... 233
Amputation for fungus hæmatodes of the breast.................... 255
Amputation, cases of.............. 477
Anatomy, Mr. Quain's elements of .. 67
Anatomy, Mr. Guthrie's letter on.... 355
Anderson, Dr. his amputation of the lower jaw .................... 567
Andry, Dr. on gangrena senilis...... 151
Aneurism of the aorta, bladder mistaken for .................... 152
Aneurism of the meningea media.... 203
Aneurismal dilatation of the posterior auris, &c. .................... 507
Angina pectoris .................... 196
Angina pectoris — coronary arteries sound.................... 573
Angina pellicularis, M. Laennec on.. 466
Annesley, Mr. on the diseases of warm climates.................... 309
Anomalous pain in the head, curious case of .................... 172
Anti-inebriants, basis of the........ 194
Anus, imperforate, case of.......... 515
Apoplexy thought to be caused by gymnastics .................... 211
Apoplexy of the lungs.............. 514
Apoplexy, chronic inflammation of the cerebral vessels.................... 585
Apothecaries' Company, new curriculum of the.................... 189
Armstrong, Dr. his morbid anatomy 118
Arsenic, apparently fatal effects of .. 170
Arterial system, general induration of the.................... 174
Artificial anus after hernia ........ 574
Ascites cured by pressure .......... 224
Ascites, apparently cured by perspiration.................... 461
Ashwell, Mr. on parturition ........ 97
Atlas, disease and dislocation of the.. 582
Atmospheric constitution of 1825-6-7 and 8 .................... 202
Averill, Mr. on loose cartilages...... 181
Auscultation, M. Laennec on ...... 420

B.

Ballingall, Dr. his report .......... 175
Barbadoes, climate of.............. 207
Bark and arsenic in neuralgia ...... 369
Bell, Mr. on diseases of the bones.... 405
Bladder mistaken for aneurism of the aorta.................... 152
Blister, nitrate of silver as a........ 449
Blisters, Dr. Kennedy on............ 564
Blood-letting, experiments on ...... 248
Blundell, Dr. his cases of amputation . 215
Boivin, Madame, on abortion ...... 257
Bone, inflammation of.............. 407
Bouillaud, M. on chronic inflammation of the cerebral vessels......... 585
Bowels and stomach, morbid anatomy of the.................... 118
Brachial artery, aneurism of the .... 540
Brain, hypertrophy of the.......... 565
Breast, Sir A. Cooper on diseases of the 381
Breast, common inflammation of the . 384
Breast, chronic abscesses in the...... 385
Breast, lacteal swelling of the ...... 386
Breast, hydatid disease of the ...... 386
Breast, chronic tumour of the ...... 392
Breast, cartilaginous tumour of the.. 394
Breast, adipose tumour of the ...... 395
Breast, large and pendulous........ 395
Breast, scrofulous swelling of the.... 396
Breast, irritable tumour of the ...... 397
Breast, ecchymosis of the .......... 398
Bright, Dr. on the intestines in fever 321
Brodie, Mr. on loose cartilages...... 525
Bronchotomy for laryngitis ........ 263
Broussaisism, Dr. Brown on ........ 90
Brown, Dr. his medical essays ...... 85
Brown, Dr. on ischuria renalis...... 254

Brown, Dr. on the mercurial treatment of fever .................... 459
Bubo treated by iodine ............ 131
Buchanan, Mr. on diseased joints.... 128
Burns and scalds .................. 237
Burrows, Dr. on insanity .......... 1

C.

Cæsarian operation, successful case of 264
Calcutta Society, transactions of the . 399
Canceroid ulcers of the lip cured, cases of ........................... 535
Cancer of the cervix uteri .......... 159
Cancer of the tongue—arsenic apparently fatal .................... 170
Cancer of the rectum, operation for.. 581
Cancerous tumours, cases of........ 226
Cardia, stricture of the ............ 120
Carlisle, Sir A. his letter in the Times 546
Carriage exercise, M. Coriolis on.... 519
Cartilaginous degeneration of bone .. 418
Cataract and glaucoma ............ 472
Cerebral affection periodical........ 195
Cervical vertebræ, spontaneous luxation of ........................ 455
Cervical vertebræ, supposed caries of the ........................... 456
Cervical vertebræ, first and second, caries of the .................. 582
Cerumen, redundancy or deficiency of 47
Cheek, remarkable enlargement of&c. 172
Chevenix, Mr. on animal magnetism . 560
Chlorate of lime, effects of.......... 222
Cholera, Dr. Christie on............ 123
Cholera, treatment of.............. 127
Chronic affections of the joints...... 178
Clavicle excised for osteo-sarcoma.... 549
Climate of Barbadoes—consumption . 207
Colchicum, effects of, on the urine .. 192
Cold air in pulmonary diseases...... 559
Compound fractures, treatment of .. 262
Compound fracture of the tibia and fibula.......................... 157
Conception, Mr. Ashwell on ........ 102
Consumption—St. John Long ...... 245
Convulsions during or after delivery . 108
Cooper *versus* Wakley.............. 268
Cooper Sir A. on diseases of the breast 381
Cranium, compound fracture of the, trephining ...................... 586
Curriculum, new, of the Apothecaries' Company ...................... 189
Cut-throat, case of—abscess in front of the windpipe ................ 575
Cut-throat—curious symptoms of cerebral disturbance .............. 172

D.

Dancing, Mr. Sheldrake on ........ 489
Dandy fever ...................... 205
Dangerous symptoms after drinking porter ......................... 530
Deafness, Mr. Stevenson on ........ 45
Death of Dr. Gall ................ 190
Death from stricture .............. 204
Delirium tremens, treatment of .... 16
Delirium traumaticum—effects of opium ........................... 525
Diarrhœa hectica. Mr. Tytler on .... 399
Difficult labours, Mr. Ashwell on.... 109
Digitalis in insanity................ 23
Diseased joints, Mr. Buchanan on.... 128
Diseases of the stomach, Dr. Abercrombie on ...................... 328
Diseases of the bones, Mr. B. Bell on . 405
Dislocation of head of humerus, under pectoral muscle ................ 138
Dislocation of the humeri undiscovered 142
Dislocation of the radius backwards . 177
Dislocation into the ischiatic notch .. 238
Dislocation into the ischiatic notch, diagnosis of .................... 251
Dislocation, partial, of the knee .... 582
Dropsy with pregnancy ............ 104
Dropsy, iatraleptic treatment of .... 199
Dupuytren, M. on fractures and dislocations ...................... 133
Dupuytren M. on the right iliac fossa 217
Dupuytren foiled in lithotomy ...... 453
Dyspepsia, Dr. Abercrombie on...... 338
Dyspnœa, acute intermittent........ 532

E.

Emphysema, singular case of........ 533
Encysted tumour of the scalp........ 173
Epidemic, mysterious, at Paris...... 156
Epilepsy cured by an attack of the Parisian epidemic.................. 460
Ergot of rye for polypus uteri........ 374
Erysipelas—effects of incisions ...... 236
Erysipelas treated by nitrate of silver 445
Erysipelas of the head and face...... 579
Examinations in medicine.......... 241
Exercise, Dr. Johnson on .......... 520
Exostosis, wonderful conversion of, into adipocere .................. 157
Extirpation of the breast, fatal results of .............................. 238
Extravasation of urine, incisions for.. 222
Extra-uterine fœtation, remarkable case of ........................ 223

F.

Face and forehead, form of.......... 506
False reports ! .................... 182
Farre, Dr. on angina pectoris ...... 196
Fasciæ of the pelvis................ 534
Femoral hernia, remarkable case of.. 574
Femoral artery and vein, wound of the, case of ........................ 589
Fever, Dr. O'Reardon on .......... 259

Fever, treatment of.................. 326
Fistulæ treated by iodine .......... 131
Flooding labour, Mr. Ashwell on .... 112
Foreign bodies in the larynx, cases of 517
Fractures and dislocations of upper extremity ...................... 132
Fracture of the upper extremity of the humerus ...................... 135
Fracture of the neck of the humerus mistaken ...................... 140
Fractures, treatment of, at Glasgow.. 165
Fractures and dislocations, cases of .. 175
Fractures of the neck of the thigh-bone 235
Fractured humerus, non-union of.... 568
Fragilitas ossium.................. 413
Freemason's Tavern—the illustrious radicals ........................ 305
French medical reform ............ 542
Fungous tumours from the gum—operation ...................... 588
Fungus hæmatodes of the breast—amputation........................ 255

G.

Gall, Dr. death of................. 190
Gangrene, traumatic, cases of, and remarks on ..................... 145
Gangrena senilis, Dr. Andry on...... 151
Gangrene of a limb after wound of its principal vessel ................ 593
Gastric and diaphragmatic perforation 221
Gastrodynia, Dr. Abercrombie on.... 340
Gibraltar epidemic ................ 564
Gonorrhœa—purulent ophthalmia .. 528
Green, Mr. his taliacotian operation.. 571
Gregory, Dr. his practice of physic .. 115
Guthrie, Mr. his letter on anatomy .. 355
Guthrie, Mr. on cataract and glaucoma 472
Guy's Hospital, resolutions of the students at........................ 305

H

Hæmorrhoids, French treatment of.. 527
Harwood, Dr. on the southern coast of England .................... 24
Haslam—his *resignation* of the presidential chair................... 496
Hastings, Dr. on the signs of pulmonary tubercles .................. 501
Heart, neuralgic and sympathetic affections of ..................... 93
Hepatic abscess—Mr. Waldron's case 515
Hepatic abscess, external pointing of 438
Hepatic abscess, opening into the lungs 440
Hepatitis, chronic, Mr. Annesley on.. 309
Hepatitis, chronic, treatment of .... 317
Hereditary predisposition to madness 11
Herniæ of children, leeches in the .. 166
Hernia—operation—difficult reduction of the gut ................... 475
Higginbottom, Mr. on nitrate of silver 444
High operation for the stone, case of 569
Hodgkin, Dr. on intermissions of the pulse ........................... 562
Hospital gangrene, cases of ........ 167
Hospital reports and elections ...... 546
Hospital gangrene, mode of treating, 589
Humerus, fracture of the neck of the 136
Hydrocele, sacculated, Mr. Guthrie's remarks on .................... 231
Hydrocele—varieties of ............ 539
Hydrocele, excision of the tunica vaginalis ......................... 238
Hydrocephalus, Dr. Mills on........ 28
Hydrocephalus, acute, description of 30
Hydrocephalus, chylopoietics, disorder in........................... 33
Hydrocephalus, diagnosis of......... 35
Hydrocephalus, treatment of ...... 37
Hydrocephalus, chronic, symptoms of 41
Hydrocephalus, chronic, treatment of 41
Hydrocephalus, prevention of ...... 43
Hypertrophy of the brain, M. Laennec on ............................ 565
Hysteria, curious case of .......... 461

I.

Iatraleptic treatment of dropsy...... 199
Iliac fossa, phlegmonous tumours in the .......................... 217
Imperforate anus—operation—death . 515
Incisions in erysipelas....... ...... 236
Induration, general, of the arterial system ........................ 174
Infirmaries and dispensaries........ 544
Inflammatory origin of tubercles .... 74
Inflammation; influence of the nervous system .................... 198
Injection of a psoas abscess ........ 153
Insanity, Dr. Burrows on .......... 1
Insanity, moral causes of .......... 3
Insanity, physical causes of ........ 6
Insanity, hereditary predisposition to . 11
Insanity, divisions of .............. 11
Insanity, characters, or symptoms of 13
Insanity, seclusion in .............. 17
Insanity, moral treatment of..... .. 18
Insanity, medical treatment of...... 19
Insurance of lives—medical evidence 213
Intemperance as a disease, Dr. Kain on 193
Intermissions of the pulse, on what depending.................... 562
Intermittent dyspnœa.............. 532
Interstitial absorption of bone ...... 413
Introduction of a bougie, followed by purulent depôts ................ 458
Iodine in diseases of the joints ...... 129
Iodine in enlargement of the uterus.. 200
Ischiatic notch, dislocation into the.. 238
Ischiatic notch, diagnosis of dislocation into the.................. 251
Ischiatic notch, dislocation into the.. 469
Ischiatic notch, dislocation into the . 537
Ischuria renalis, Dr. Brown on...... 254

J.
Johnson, Dr. his case of pneumothorax ........................ 478

K.
Kay, Dr. his experiments on tubercles, 81
Kennedy, Dr. on blisters ........... 564
Knee, neuralgia of the ............ 57
Knee, partial dislocation of the ..... 582

L.
Labour, varieties of................ 105
Laennec, M. on diseases of the chest, 420
Laennec, Dr. on hypertrophy of the brain ........................ 565
Lambert and the medical societies of London ........................ 493
Lambert, Mr. his accuracy ......... 563
Laryngitis—bronchotomy successful, 263
Larynx, foreign bodies in the; bronchotomy ........................ 517
Lazaretto in Harley Street ........ 246
Leamington waters, analysis of ..... 27
Libel, age of...................... 266
Ligature of the femoral artery and vein ........................ 591
Lip, ulcers of, cured .............. 535
Lithontripteur.................... 191
Lithontripteur used without success, 454
Lithotomy and lithotrity .......... 69
Lithotomy, mode of operating ...... 231
Lithotomy—M. Dupuytren foiled.... 453
Lithotomy, cases of................ 465
Lithotomy, difficult case of ........ 538
Liver and lungs, purulent depôts in.. 169
Liver, acute diseases of the ........ 344
Liver, chronic diseases of the ....... 347
Liver, Mr. Annesley on abscess in the, 435
London University, opening of the .. 184
Loose substances removed from the knee ........................ 181
Loose cartilage in the knee-joint.... 524
Loudon, Dr. on the Leamington Spa, 24
Lower extremity, swelling of, post febrem ........................ 180
Lower jaw, amputation of the ...... 557
Lumbar abscess in a horse.......... 243

M.
Macculloch, Dr. on neuralgia ...... 49
Macculloch, Dr. on neuralgia ...... 357
Macfarlane, Dr. on polypus uteri.... 373
Maclachlan, Dr. his hospital report.. 586
Mænorrhagia produced by mercury, 471
Malaria, Dr. Brown on ............ 87
Malden, Dr. on inflammation ...... 198
Malvern waters, Mr. Addison on the, 24
Mayo, Mr. his curious case ........ 234
Mayo, Mr. on varicose veins........ 250
Meatus auditorius, foreign bodies in the ........................ 573
Medical treatment of insanity ...... 19
Medical essays, Dr. Brown's ........ 85
Medical evidence in courts of justice, 213
Medical examinations.............. 241
Medical reporting ................ 249
Medical degradation .............. 493
Medical justice and liberality ....... 498
Medical reform .................... 542
Medical societies, reporting from.... 543
Meningea media, aneurism of the ... 203
Menses, absence of the ............ 585
Menstrual secretion, nature of...... 99
Mercurial treatment of fever, Dr. Brown, on ........................ 459
Mercury, injection of producing tubercles ........................ 81
Mercury in fungus testis ........... 465
Mesmerism, Mr. Chevenix on ....... 560
Metallic tinkling, Dr. Williams on the, 581
Milligan, Dr. on the face and forehead 506
Mills, Dr. on hydrocephalus ........ 28
Morbid anatomy of the intestines, Dr. Bright on ........................ 321
Morbid phenomena, succession of, in one individual ................ 460
Morbus coxarius, Dr. Ballingall on .. 179
Mott, Valentine, his excision of the clavicle ........................ 549
Mucous membrane of the stomach, &c. natural state of ............ 124
Muscæ volitantes, pathology of...... 512

N.
Napoleon murdered by the English Government .................. 425
Napoleon à Sainte Helene .......... 424
Napoleon, account of his illness .... 427
Napoleon, dissection of ............ 432
Neck of the thigh-bone, interstitial absorption of .................. 414
Neuralgia, Dr. Macculloch on ...... 49
Neuralgiæ, in various parts of the body ........................
Neuralgia from injuries ............ 62
Neuralgia, remarks on the medical case of ........................ 212
Neuralgia, Dr. Macculloch on ...... 357
Neuralgia ophthalmica—rheumatism of the eye ...................... 357
Neuralgia and intermittent, connexion between...................... 365
Neuralgia, cure of ................ 368
Neuralgia, oil of turpentine for .... 505
Neuralgia, curious case of.......... 553
Nitrate of silver, Mr. Higginbottom on, 444
Nitrate of silver in diseases of the eye, 225
Nitro-muriatic bath, directions for using ........................ 318
Non-union of fractures ............ 568
Non-united fractures, treated by iodine ........................ 132

O.

Odour, peculiar, of insane persons .. 15
Operation for pneumo-thorax ....... 478
Operation for hepatic abscess ...... 439
Opium in the delirium traumaticum . 525
Optic nerve, neuralgia of .......... 56
O'Reardon, Dr. on fever ........ .. 259
Organic diseases of the stomach .... 336
Œsophagus, stricture of the ........ 119
Osteo-sarcoma, excision of the clavicle for ........................ 549
Ovary, encysted tumour of, cured.... 467

P.

Pancreas, pathology of the.......... 353
Paraplegia, use of strychnine in ..... 164
Parisian epidemic .................. 156
Parturition, Mr. Ashwell on ........ 97
Par vagum, neuralgia of the ........ 553
Patella, remarkable alteration of the, 556
Pathology of muscæ volitantes ...... 512
Pecchioli, Dr. his lithontripteur .... 191
Penis, suicidal amputation of the.... 539
Perforation of the stomach and diaphragm ......................... 221
Perineum, contusion of ............. 534
Perineum, extensive laceration of the, 538
Periodical head-ache .............. 52
Periodical cerebral affection ........ 195
Periodical vino-mania............... 253
Periosteum, diseases of the ........ 407
Phlebitis, curious instance of........ 468
Phlegmatia dolens in a male........ 526
Phlegmonous tumours in the right iliac fossa ...................... 217
Phthisis pulmonalis in India........ 401
Physic, Dr. Gregory's elements of .. 115
Physical signs of diseases of the lungs, Dr. Williams on ................ 450
Pin-prick, followed by extensive suppuration ....................... 552
Pleximeter, M. Piorry's............ 421
Pneumo-thorax, case of ............ 456
Pneumo-thorax in a medical gentleman ......................... 478
Pneumo-thorax, observations on .... 482
Popliteal aneurism, case of, and remarks on ...................... 228
Polypus uteri, case of.............. 227
Polypus of the uterus, Dr. Macfarlane on .......................... 373
Polypus of the uterus, with pregnancy 375
Polypus, malignant, of the uterus.... 380
Porter, dangerous symptoms from drinking ....................... 530
Posterior tibial artery, aneurism of the........................... 469
Prepuce, removal of, followed by pneumonia ....................... 577
Pressure for ascites................ 224
Professional or unprofessional ?...... 499
Psoas abscess, injection of its cavity.. 153
Puerperal peritonitis, Mr. Ashwell on, 113
Puerperal peritonitis, rapidly fatal .. 201
Pulmonary tubercles, signs of ...... 501
Pulmonary apoplexy, case of........ 514
Pulsating tumours of the scalp, cases of, with remarks................ 507
Punctured wounds treated by nitrate of silver......................... 447
Purulent ophthalmia following gonorrhœa ........................... 528
Pylorus, stricture about the ........ 120

Q.

Quack medicines—French regulations on ............................. 254
Quain, Mr. his elements of anatomy, 67

R.

Radius, dislocation of, forwards and upwards ....................... 240
Ranula, treated by the seton......... 578
Rectum, neuralgia of the .......... 59
Rectum, scirrho-contracted, curious case of ........................ 208
Rectum, operation for cancer of the, 581
Reporting, medical, remarks on .... 249
Reporting from medical societies.. .. 543
Respiration of cold air in pulmonary diseases ....................... 559
Retention of urine in pregnancy .... 103
Rheumatism, metastases of ........ 92
Rheumatism of the eye, Dr. Macculloch on ........................ 357
Rheumatism of India, Mr. Henderson on .......................... 403

S.

Saint Helena, Napoleon at, by M. Hereau ........................ 425
Saint Helena, topography of........ 425
St. John Long's quackery .......... 245
Salivary fistula, treatment of........ 226
Saphena vein, division and excision of, 162
Scalp, encysted tumours of the...... 173
Scalp-wound—erysipelas of the head and face ...................... 579
Scarlett, Sir James, his admirable speech ......................... 280
Sciatica, Dr. Macculloch on ........ 60
Scirrhus of the male breast ......... 472
Secondary hæmorrhage, remarkable kind of ........................ 470
Sheldrake on dancing............... 489
Sheldrake, Mr. on personal appearance ......................... 554
Sigmoid flexure of the colon, stricture of ........................ 95
Southern Coast of England, Dr. Harwood on ....................... 24
Spina ventosa, Mr. Bell on ......... 416
Spinal neuralgia, instructive case of, 244
Spinal disease, unsuspected ........ 462

Spine tinker, the ................ 256
Spleen, pathology of the............ 351
Sloughing ulcers, Mr. Langstaff on .. 404
Stedman, Dr. on the dandy fever of Santa Cruz .................... 205
Stevenson, Mr. on deafness ........ 45
Stomach, scirrhus of the .......... 121
Stomach and duodenum, severe affection of the .................... 211
Stomach, organic diseases of the .... 336
Stomach, functional diseases of the.. 338
Stomach, Dr. Abercrombie on the diseases of the .................... 328
Stomach, inflammation and ulceration of the......................... 330
Stone in the bladder, high operation for ............................ 569
Strangulated inguinal hernia........ 234
Stricture, death from .............. 204
Strychnine, use of, in paraplegia.... 164
Suicidal and infanticidal mania...... 474
Suppuration, extensive, after the prick of a pin...................... 552
Surgical reform .................. 500
Syme, Mr. his case of aneurismal dilatation......................... 507
Synovitis, Dr. Couper on .......... 457

T.

Taliacotian operation, Mr. Green's case........................... 571
Taliacotian operation.............. 588
Taraxacum, good formula for ...... 320
Tibia and fibula, compound fracture of the......................... 154
Tic douloureux, Dr. Macculloch on .. 49
Tic douloureux, description of ...... 50
Tooth-ache, Dr. Macculloch on...... 63
Toxicological considerations on bloodletting ......................... 248
Transactions of the Medical Society of Calcutta ....................... 399
Traumatic gangrene, cases of, and remarks on ...................... 145
Traumatic tetanus, pathology of .... 261
Travelling, Dr. Johnson on ........ 520
Travers, Mr. his curious case of dislocation ........................ 238
Trial of Cooper *versus* Wakley...... 268
Trial of Cooper *versus* Wakley, commentary on ...................... 297
Trismus from cold, cure of ........ 468
Tubercles, Dr. Alison on .......... 72
Tubercles, inflammatory origin of .. 74
Tubercles, prevalence of in particular classes ....................... 80
Tubercles, hereditary .... .......... 75
Tubercles of the lungs, signs of..... 501
Tubercles, Medicus on ............ 513
Tumour growing from the uterus ... 229
Tumour in the orbit—abscess in the brain ........................... 470
Turpentine, oil of, in sciatica ....... 505
Tweedie, Dr. on swelling of the lower extremity ...................... 180

U.

Ulcerated prepuce cured by the chlorate of lime .................... 222
Ulceration of the stomach.......... 330
Ulceration of the scrotum—fungus testis, case of .................. 465
Ulcers, treatment of .............. 167
Ulcers of the bowels .............. 323
Unguentum argent. nit. in diseases of the eye ........................ 225
Ununited fracture—ends of bone excised ......................... 176
Urinary abscess, treatment of ...... 168
Uterus, M. Lisfranc on amputation of the neck of ................. 159
Uterus, enlargement of, treated by iodine ......................... 200
Uterus, amputation of the........... 215
Uterus, tumour of the.............. 229
Uterus, fibrous polypus of the ...... 373

V.

Valvular obstruction of the rectum .. 208
Varicose ulcers, treatment of ...... 162
Varicose veins, treatment of ........ 250
Varix of the saphena, simulating hernia ......................... 575
Veins, purulent concretions in the .. 468
Vertebræ, displacement of ......... 166
Vino-mania, periodical ............ 253
Visceral depôts, after wounds, &c. .. 169
Visceral neuralgia in a medical gentleman ....................... 487

W.

Walking—Mr. Sheldrake's discoveries, 554
Warm climates, Mr. Annesley on the diseases of ..................... 309
Williams, Dr. on auscultation ...... 450
Wound of the heart—pericarditis ... 463
Wound of the femoral artery and vein ......................... 589
Wound of the femoral vessels in the sheath of the triceps, Mr. Johnson's case ........................... 594

